# Prior Methamphetamine Use Disorder History Does Not Impair Interoceptive Processing of Soft Touch in HIV Infection

**DOI:** 10.3390/v13122476

**Published:** 2021-12-10

**Authors:** Amanda Bischoff-Grethe, Ronald J. Ellis, Susan F. Tapert, Martin P. Paulus, Igor Grant, Translational Methamphetamine AIDS Research Center (TMARC)

**Affiliations:** 1 Department of Psychiatry, University of California, San Diego 9500 Gilman Drive, MC 0738 La Jolla, San Diego, CA 92093, USA; stapert@health.ucsd.edu (S.F.T.); igrant@health.ucsd.edu (I.G.); 2 Department of Neurosciences, University of California, La Jolla, San Diego, CA 92093, USA; roellis@health.ucsd.edu; 3 Laureate Institute for Brain Research, Tulsa, OK 74136, USA; mpaulus@laureateinstitute.org

**Keywords:** touch, insula, methamphetamine dependence, HIV infection

## Abstract

Introduction: Interoception, defined as the sense of the internal state of one’s body, helps motivate goal-directed behavior. Prior work has shown that methamphetamine (METH) use disorder is associated with altered interoception, and that this may contribute to risky behavior. As people with HIV (PWH) may also experience disrupted bodily sensations (e.g., neuropathy), an important question is whether PWH with a history of METH use disorder might exhibit greater impairment of interoceptive processing. Methods: Eighty-three participants stratified by HIV infection and a past history of methamphetamine use disorder experienced a soft touch paradigm that included slow brush strokes on the left forearm and palm during blood-oxygen level-dependent functional MRI acquisition. To assess differences in interoception and reward, voxelwise analyses were constrained to the insula, a hub for the evaluation of interoceptive cues, and the striatum, which is engaged in reward processing. Results: Overall, individuals with a history of METH use disorder had an attenuated neural response to pleasant touch in both the insula and striatum. Longer abstinence was associated with greater neural response to touch in the insula, suggesting some improvement in responsivity. However, only PWH with no METH use disorder history had lower brain activation in the insula relative to non-using seronegative controls. Conclusions: Our findings suggest that while METH use disorder history and HIV infection independently disrupt the neural processes associated with interoception, PWH with METH use disorder histories do not show significant differences relative to non-using seronegative controls. These findings suggest that the effects of HIV infection and past methamphetamine use might not be additive with respect to interoceptive processing impairment.

## 1. Introduction

The use of methamphetamine (METH) among individuals at risk for HIV transmission has been called a “double epidemic” [1]; it significantly contributes to HIV transmission by enhancing risky behavior [2], and it may be associated with delays in HIV diagnosis and treatment initiation [3]. More important, its continued use in people with HIV (PWH) has been associated with reduced effectiveness of antiretroviral treatment [4] and higher plasma viral load [5,6], possibly due to poor medication adherence [5]. Independently, both HIV infection and METH can alter brain structure [7,8] and function [9,10,11,12], yet very little is known about their interaction. Some neuroimaging studies suggest there is an additive effect, whereby METH exacerbates the effects of HIV infection on brain metabolites [13] or cerebral blood flow [14]. Alternatively, structural neuroimaging studies report that METH use disorder in PWH may provide a mitigation effect [15], as it does not significantly moderate measures of cortical surface area, volume, or thickness [16]. Only one functional neuroimaging study to date has examined the effects of METH use disorder with HIV infection and determined that METH may mitigate the effects of HIV infection on striatal activation during a motor switching task [17]. Thus, despite the relative prevalence of METH use in PWH, little is known about their interaction on brain function.

Interoception, defined as the sense of the physiologic condition of the body, is an important process for maintaining homeostasis [18]. These bodily sensations, such as pain [19], temperature [20], and sensual touch [21,22], are carried by unmyelinated primary afferent C-fibers and involve a distinct neural pathway that includes the lateral spino-thalamic tract, midbrain homeostatic nuclei, the ventromedial thalamus and the posterior insular cortex [23]. These signals are later integrated within the anterior insula cortex [18], which is integrally connected with subcortical [24], limbic [25], and executive control brain systems [26], enabling an individual to assess internal sensations and act upon them. Notably, the ventral anterior and middle insula process social-emotional and sensorimotor information [27], enabling the integration of interoceptive stimuli with an emotional response that will lead to an action or decision [28], suggesting that interoception may have a profound impact on cognitive decision making.

Altered interoceptive neural processing may impair the interpretation of and response to affective stimuli. Prior work in individuals with past METH use disorder histories has demonstrated an attenuated response in the insula to touch anticipation and receipt relative to healthy individuals [29], suggesting that METH may disrupt interoceptive processing. Additionally, the striatum in individuals with prior METH use disorder exhibited lower response to touch anticipation but higher response to touch receipt relative to healthy individuals, demonstrating that reward processing was also perturbed. METH use also affects the release of dopamine and serotonin [30], both of which are integral to the affective touch system [31], as well as dysregulates glutamate transmission within the prefrontal cortex and insula [32,33]. Altered interoception associated with METH may thus be an important process underlying changes in risky and reward-related behaviors.

HIV infection is also associated with decreased dopamine levels [34] and glutamate-evoked toxicity [35], and this may be associated with distal neuropathic pain in PWH [36]. Pro-inflammatory cytokines in the peripheral system may also contribute to distal neuropathic pain through stimulation of unmyelinated C-fibers [37,38,39]. Recent evidence also suggests that the insula may be influenced by peripheral inflammation [40,41], thereby disrupting interoceptive signaling and associated cognitive processes. Importantly, peripheral neuropathy in PWH can persist despite viral suppression with antiretroviral therapy, even in individuals without prior exposure to neurotoxic nucleoside antiretrovirals [42]. Studies have previously reported reduced sensitivity to vibration in PWH [43]; however, no study to date has examined pleasant touch, which also activates C-fibers [44]. Given that allodynia is also a symptom of distal sensory polyneuropathy [45], pleasant touch in PWH could be attenuated or even feel painful. Other studies have reported that substance use increased the risk of distal sensory polyneuropathy in PWH [46]. Given that METH use is associated with an increased risk of HIV infection, their comorbidity could be associated with significantly altered interoceptive processing.

This study is the first to examine whether PWH exhibit an altered interoceptive brain response to pleasant touch, and whether a history of METH use disorder is linked to greater impairment in interoceptive processing that could support exaggerated reward-seeking and risk-taking behaviors seen in comorbid METH dependence and HIV infection. While prior work has established altered insula and striatal response to affective touch in relation to METH use, this has yet to be demonstrated in PWH, nor is it well understood whether PWH with METH use histories will show further attenuation of neural responsivity. A soft touch task previously shown to activate the insula and striatum probed neural response to a pleasant interoceptive stimulus [29]. We hypothesized that, relative to healthy comparison individuals, both PWH and those with METH use disorder histories would show attenuated activation within the insula and striatum in response to the anticipation of and stimulation by pleasant touch. If the effects of METH use disorder and HIV infection are additive, PWH with METH use disorder histories would show further attenuation in the insula and striatum to the anticipation and receipt of pleasant touch relative to the other cohorts. Finally, we hypothesized that a lower neural response to touch would be associated with poorer clinical measures in both PWH and individuals with prior METH use disorder.

## 2. Materials and Methods

### 2.1. Participants

Eighty-seven participants were recruited through the Translational Methamphetamine AIDS Research Center, distributed across four groups: PWH with no history of METH use disorder (HIV+/METH−, N = 20), seronegative controls with a history of METH use disorder (HIV−/METH+, N = 20), PWH with histories of METH use disorder (HIV+/METH+, N = 18), and community controls with neither of the risk factors (HIV−/METH−, N = 29). HIV status was confirmed by MedMira Multiplo rapid test (MedMira Inc., Halifax, NS, Canada). All participants were seronegative for Hepatitis C virus (HCV) as determined by the MedMira Multiplo rapid test. Current CD4 T lymphocyte counts (cells/mL) were determined by flow cytometry at a Clinical Laboratory Improvement Amendments (CLIA), or equivalent, certified medical center laboratory. HIV RNA levels were measured in plasma by reverse transcriptase PCR (Roche Amplicor, v. 1.5, lower limit of quantitation 50 copies/mL). CD4 nadir was obtained by self-report, with confirmation by documented prior measurements in a subset of individuals.

All METH+ participants met diagnostic (DSM-IV) [47] criteria for lifetime amphetamine dependence including abuse or dependence in the past 18 months as determined by the Composite International Diagnostic Interview (CIDI) [48]. METH− participants did not meet criteria for lifetime or current methamphetamine abuse or dependence. Exclusionary criteria for all groups were DSM-IV criteria for: alcohol dependence in the past year, other substance dependence in the last 5 years, other substance abuse (e.g., cocaine, opioids) within the last year, and remote (i.e., >5 years) but significant history of alcohol or other drug dependence. Given the high comorbidity of alcohol and marijuana abuse and marijuana dependence with methamphetamine use, METH+ individuals with such histories were not excluded.

Participants also were excluded for: positive urine toxicology screen or Breathalyzer for illicit drugs (other than marijuana due to its long-lasting detectability) or recent drinking on the day of scan; MRI contraindication; lifetime history of schizophrenia or other psychotic disorder; previous cerebrovascular events as determined by comprehensive neurological exam; head injury with loss of consciousness >30 min or neurologic complications; demyelinating diseases; or seizure disorder. Participants were recruited from the San Diego area via flyers and advertisements at community events and drug dependence treatment programs. All participants gave written consent prior to enrollment and again prior to scanning. The University of California, San Diego Human Research Protections Program approved all procedures.

### 2.2. Measures

As part of a larger assessment battery, participants completed the Wide Range Achievement Test-4 as a measure of premorbid intelligence and quality of education (WRAT-4) [49] and a comprehensive neuropsychological test battery [50]. A neurological exam was also included to assess for signs of clinical (bilateral distal vibration, sharp, and touch loss) and self-report symptoms (paresthesias, pain, numbness) of neuropathy, defined as 1 or more signs of a diminished ability to recognize vibration and reduced sharp-dull discrimination. On the scan day, participants also completed the Beck Depression Inventory (BDI-II) [51].

### 2.3. Imaging Procedures

#### 2.3.1. Soft Touch Paradigm

A soft boar bristle brush (OXO International Ltd., New York, NY, USA) was administered on 4 cm long regions of skin by a trained research assistant. For a given trial, stimulation occurred on either the ventral surface of the left forearm, a region believed to contain dense mechano-receptive C-fibers, and on the palm, where these fibers are absent [21,22,52]. These regions were both pre-measured and pre-marked for consistency, and each soft brush stroke occurred at a velocity of 2 cm/s in a proximal to distal direction, standardized by an audio tone that was routed only to the research assistant’s headphones [29,53,54]. This velocity has been previously shown to activate the posterior insula, and it is within the optimal range (1–10 cm/s) for C-fiber stimulation [52]. The force applied was equal to the brush’s weight.

**Figure 1:**
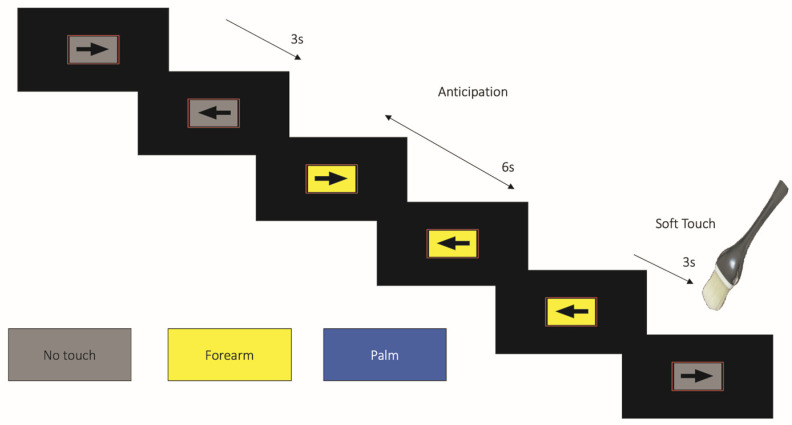
The soft touch continuous performance task. Participants anticipated or experienced a soft touch via controlled brush strokes on either the forearm (indicated by a yellow background) or palm (indicated by a blue background).

Participants performed two functional runs of the task as part of a larger functional neuroimaging battery (Figure 1). During each functional run, participants were presented with a left or rightward pointing arrow on a gray rectangular background for 3 s. Participants were asked to quickly respond by pressing either the left or right button of a button box, with the direction corresponding to the direction of the arrow. The background of the arrow would change color to indicate one of three conditions: (1) the baseline condition (gray background), in which no stimulus was expected or administered, and averaging 9 s (three consecutive arrow trials) in duration; (2) anticipation of soft touch of the left forearm (yellow background), lasting 6 s, and which indicated the participant could expect a soft touch of the forearm; and (3) anticipation of soft touch of the left palm (blue background), also lasting 6 sec, indicating the participant should expect a soft touch of the palm. Following the anticipatory periods, the soft touch condition would occur for 3 s, whereby the brush was applied to the previously indicated location for the first 2 s of the trial. Overall, anticipation and soft touch occurred twenty times for each location (palm, forearm).

Response accuracy and reaction time were recorded for all trials. Following the neuroimaging session, participants also completed a visual analog scale (VAS) questionnaire to independently rate pleasantness, unpleasantness, and intensity of the soft touch of the forearm and palm, from “0—not at all” to “10—extremely.”

#### 2.3.2. Image Acquisition

Data were acquired using T2* weighted echo planar imaging (EPI) on one of two scanning systems: a 3T General Electric Signa HDx (Milwaukee, WI, USA) or (252 volumes, TR = 2 s, TE = 30 ms, flip angle = 90°, FOV 24 cm, 64 × 64 matrix, 3.55 × 3.55 mm in-plane resolution with 40 3.0 mm (2.6 mm + 0.3 mm gap) ascending interleaved axial slices) or a 3T General Electric Discovery MR 750 (Milwaukee, WI, USA) (identical parameters as above except 3.75 × 3.75 mm in-plane resolution, 40 3.0 mm ascending interleaved axial slices). To permit activation localization and spatial normalization, the following were acquired: High-resolution T1-weighted FSPGR anatomical images (Signa HDx: flip angle = 8°, 256 × 256 matrix, 172 1 mm sagittal slices, TR = 7.77 s, TE = 2.97 ms, and 0.97 × 0.97 mm in-plane resolution; for MR 750, same as above except TR = 8.1 s, TE = 3.17 ms, 1 × 1 mm in-plane resolution). EPI-based field maps corrected susceptibility-induced geometric distortions. The gradients system and application were not changed during the scanner upgrade, and all post processing and analysis steps were consistent across datasets. Multisite imaging studies suggested that inter-participant variance far outweighs that of site or magnet variance [55]. There were no differences in HIV or METH status, or in other demographic factors based on scanning system employed (see Table 1).

#### 2.3.3. Image Preprocessing

Functional images were preprocessed using Analysis of Functional NeuroImages (AFNI) [56] and FSL [57]. EPIs were slice-time corrected, motion-corrected, and aligned to high-resolution anatomical images using AFNI’s align_epi_anat.py [58]. Movement parameters were visually inspected for extensive motion exceeding 3 mm, and one HIV+/METH+ dataset was subsequently excluded. Time points with isolated head movements not corrected by coregistration were censored. T1-weighted images were skull-stripped using FreeSurfer’s mri_watershed [59] and registered to the MNI-152 atlas using affine transform followed by nonlinear refinement via FSL’s FLIRT and FNIRT [60,61]. Functional data were aligned to standard space, resampled to 3 mm isotropic voxels, and smoothed to a 6 mm FWHM using AFNI’s 3dBlurToFWHM. For each participant, AFNI’s 3dDeconvolve was used to determine activation related to the soft touch paradigm. Four task regressors (anticipation forearm, anticipation palm, soft touch forearm, soft touch palm) were convolved with a modified hemodynamic response function. Six motion regressors and first-, second-, and third-order polynomial trends were included as covariates of no interest. Following deconvolution, the four task-based beta regressors were converted to percent signal change.

### 2.4. Data Analysis

#### 2.4.1. Behavioral Analysis

Response accuracy and reaction time of each button press were recorded from the onset of arrow presentation. Group level statistical analyses were performed in R (http://www.r-project.org, version 3.6.0, accessed on 26 April 2019) using a linear mixed effects (LME) model, from R’s nlme package [62]. The model HIV × METH × Condition (anticipation, soft touch) × Location (palm, forearm), with Condition and Location as within-subjects factors, was used to examine both response accuracy and reaction time. The VAS predictors for “pleasantness,” “unpleasantness,” and “intensity” were analyzed as dependent measures using linear mixed effects to test for group differences in subjective reports. For all models, post hoc analyses were performed using R’s *emmeans* to computer linear contrasts [63], the *p*-values were adjusted using the False Discovery Rate (FDR) [64], and standardized effect sizes were reported.

#### 2.4.2. Regions of Interest

Regions of interest (ROIs) were derived from the Harvard-Oxford atlas [65]. Two bilateral ROIs were defined: an insula ROI, which contained the insula in its entirety, and a striatum ROI that included the caudate, putamen, and nucleus accumbens. These two ROIs were used as search regions for all group level fMRI analyses.

#### 2.4.3. Neuroimaging Analysis

Group level statistical analyses were performed using the nlme package in R (http://www.r-project.org, version 3.6.0, accessed on 26 April 2019) to assess differences in blood oxygen level dependent (BOLD) response. Data were analyzed using an HIV × METH × Condition (anticipation, soft touch) + Location (palm, forearm) linear mixed effects approach. Location was treated as a covariate, as prior studies have not demonstrated significant interactions with location [29,53,66,67]. For all analyses, subject was nested within scanner and treated as a random effect, with HIV, METH, Condition, and Location as fixed effects. Intrinsic smoothness was estimated using the spatial autocorrelation function (acf) option in AFNI’s 3dFWHMx. Minimum cluster sizes were calculated with AFNI’s 3dClustSim in order to guard against false positives. For ROI analyses, a peak voxel of *p* < 0.001 with a cluster threshold of *α* < 0.025 was required for significance. This approach employs non-Gaussian models and spatial autocorrelation functions and is more robust than traditional methods [68]. A minimum cluster size of 108 μL (4 contiguous voxels) each was required for the insula and the striatum for significance. An exploratory whole brain analysis examined group differences in activation across the whole brain (peak voxel *p* < 0.001, cluster threshold of *α* < 0.05, minimum cluster size 324 μL [12 contiguous voxels]) and is presented in the supplement (Supplemental Table S2). As with the behavioral data, R’s *emmeans* was used for post hoc analyses of significant clusters, and standardized effect sizes (ES) were reported.

#### 2.4.4. Primary Robust Regression Analyses

Within-group Huber robust regressions were conducted in R to examine the relationship of clinical variables related to METH use history (age of first use, days since last use, METH use density (total quantity/total days)) within METH+ participants, and to HIV infection (illness duration in months, current CD4, nadir CD4) within HIV+ participants. Measures were natural log transformed and z-scored prior to regression. Individual regressions were performed against the mean percent signal change for anticipation palm, anticipation forearm, soft touch palm, and soft touch forearm. Significant clusters were determined within regions of interest using AFNI’s 3dClustSim for small volume correction with a peak voxel of *p* < 0.01. Results were Bonferroni corrected for the number of measures applied to each group, two ROIs, and four conditions (*α* < 0.0021).

## 3. Results

### 3.1. Participant Characteristics

Two participants (1 HIV−/METH−, 1 HIV−/METH+) were excluded due to problems with data acquisition, 1 HIV+/METH+ participant was excluded for psychosis, and 1 HIV+/METH+ participant was excluded for motion, leaving a final sample of 83 participants (28 HIV−/METH−, 19 HIV−/METH+, 20 HIV+/METH−, 16 HIV+/METH+). Study groups did not differ on sex, handedness, or ethnicity (Table 1). The four groups did not differ significantly with respect to age, and no HIV by METH interaction was seen. Participants with METH use disorder history (HIV−/METH+, HIV+/METH+) were significantly less educated than non-dependent participants, *F*(1, 79) = 5.74, *p* = 0.02, $\eta_{p}^{2}$ = 0.068. Although there was no main effect of either METH use disorder or HIV diagnosis on WRAT-4 standard scores, the interaction of HIV with METH was significant, *F*(1, 79) = 6.13, *p* = 0.015, $\eta_{p}^{2}$ = 0.072, with the HIV−/METH− and HIV+/METH+ groups scoring higher than the other two groups. The HIV+/METH− and HIV+/METH+ groups reported greater frequencies of distal symmetric polyneuropathy (*p* = 0.013), loss of sensation (*p* = 0.004), and paresthesia (*p* = 0.003). Groups did not differ on reports of neuropathic pain (*p* = 0.11) or dysesthesia (*p* = 0.11). There was a main effect of METH use disorder history on the BDI-II, such that participants with METH use disorder history scored higher than those with no prior METH use disorder history, *F*(1, 76) = 18.69, *p* < 0.001, $\eta_{p}^{2}$ = 0.20. Individuals with METH use histories tended to have higher frequencies of most substance use disorders, as well as lifetime antisocial personality disorder, whereas all but the HIV−/METH− group had a higher frequency of lifetime major depressive disorder (Supplemental Table S1).

The HIV+/METH− and HIV+/METH+ groups did not significantly differ on infection duration ($\eta_{p}^{2}$ = 0.02), current CD4 count ($\eta_{p}^{2}$ = 0.03), or nadir CD4 count ($\eta_{p}^{2}$ = 0.01). The HIV−/METH+ and HIV+/METH+ groups did not significantly differ on age of first use ($\eta_{p}^{2}$ < 0.01), total days used ($\eta_{p}^{2}$ < 0.01), days since last use ($\eta_{p}^{2}$ < 0.01), total quantity ($\eta_{p}^{2}$ = 0.05), or use density ($\eta_{p}^{2}$ = 0.06).

### 3.2. VAS Scales

There were no significant effects of HIV status, METH status, or their interaction for VAS ratings in relation to pleasantness or unpleasantness of soft touch to either the palm or forearm, or of the intensity of soft touch to the palm, *ps* > 0.06. However, there was a main effect of HIV on the VAS ratings in relation to intensity of soft touch to the forearm, *F*(1, 79) = 5.02, *p* = 0.028, ES = 1.93, such that PWH rated touch as more intense than HIV− participants.

### 3.3. Behavioral Analyses

There were no significant main effects of HIV diagnosis, METH use disorder history, Condition, or Location with respect to correct responses, nor were any interactions significant (all *ps* > 0.055). There was a main effect of Condition, *F*(1, 237) = 25.77, *p* < 0.001, ES = 4.00, with participants responding more slowly during soft touch receipt than during anticipation. There was also an HIV × METH × Condition interaction, *F*(1, 237) = 6.05, *p* = 0.016. Post hoc analyses revealed that the HIV−/METH− had slower reaction times during soft touch receipt relative to anticipation, *t*(237) = 2.91, *p* = 0.023, ES = 3.77. This relationship was also seen for the HIV+/METH+ group, *t*(237) = 4.67, *p* < 0.001, ES = 8.00. No other significant main effects or interactions were detected.

### 3.4. Region of Interest Analyses

#### 3.4.1. Main Effect of Condition

There was a main effect of Condition across all participants in the bilateral insula and striatum in their entirety, with an increased BOLD response to soft touch receipt relative to anticipation (Table 2).

#### 3.4.2. HIV × Condition Interaction

**Figure 2:**
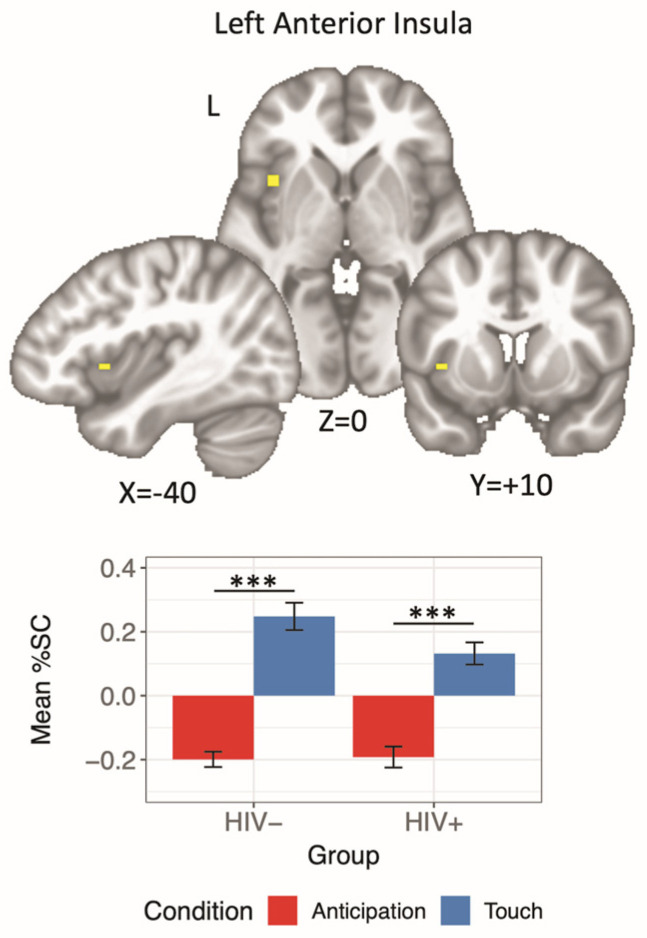
Bar plot showing a significant HIV × Condition interaction in the left anterior insula. Both HIV− and HIV+ participants exhibited a greater BOLD response to pleasant touch relative to anticipation. Error bars represent standard error of the mean. %SC: percent signal change. *** *p* < 0.001.

An HIV × Condition interaction was detected within the left insula; however, post hoc analyses suggested that participants, regardless of HIV status, showed an elevated response to soft touch receipt relative to anticipation (Table 2, Figure 2).

#### 3.4.3. METH × Condition Interaction

**Figure 3:**
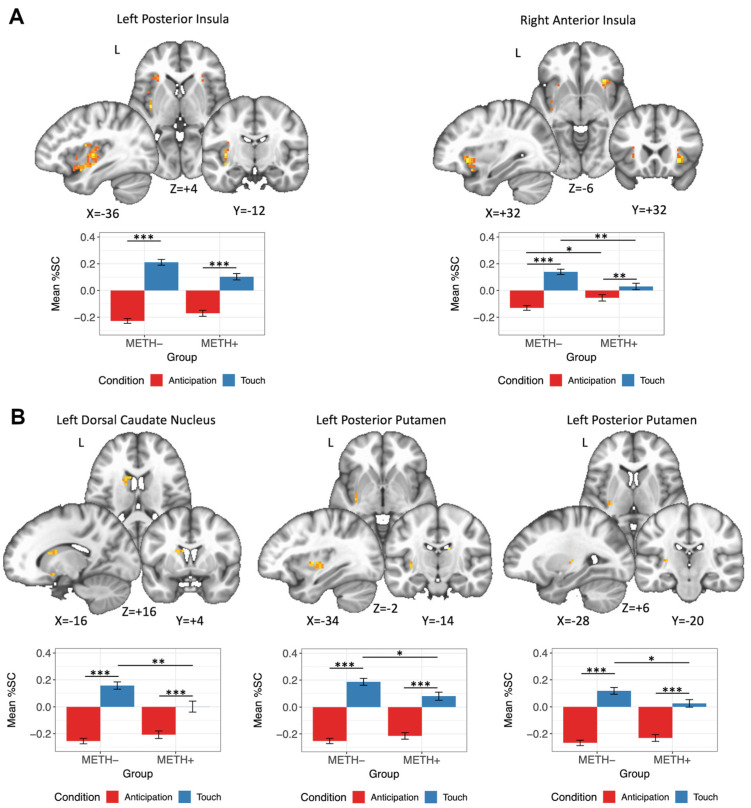
Bar plot showing a significant METH × Condition interaction with several clusters. (**A**) Within the left posterior insula and right anterior insula, both the METH− and METH+ groups demonstrated a greater BOLD response to touch receipt relative to anticipation. Within the right anterior insula, the METH+ group showed a greater BOLD response to anticipation relative to the METH− group; in contrast, the METH− group demonstrated greater BOLD response to touch receipt relative to the METH+ group. (**B**) Across all three striatal clusters, both METH− and METH+ groups showed greater BOLD response to touch receipt relative to anticipation, and the METH− group had greater BOLD response to touch receipt relative to METH+. %SC: percent signal change. * *p* < 0.05; ** *p* < 0.01; *** *p* < 0.001.

There were several clusters, predominately within the left insula, left dorsal caudate, left putamen, and left ventral striatum, demonstrating a METH × Condition interaction. In all clusters, post hoc analyses demonstrated that both METH− and METH+ individuals showed a greater response to soft touch receipt relative to touch anticipation (Table 2, Figure 3). However, clusters within the left posterior insula, the right anterior insula, and within the left dorsal caudate and posterior putamen additionally revealed that the BOLD response to soft touch receipt in METH+ individuals was attenuated relative to METH− individuals (Figure 3). METH+ individuals also had relatively greater BOLD response than METH− individuals to soft touch anticipation within the right anterior insula.

#### 3.4.4. HIV × METH × Condition

**Figure 4:**
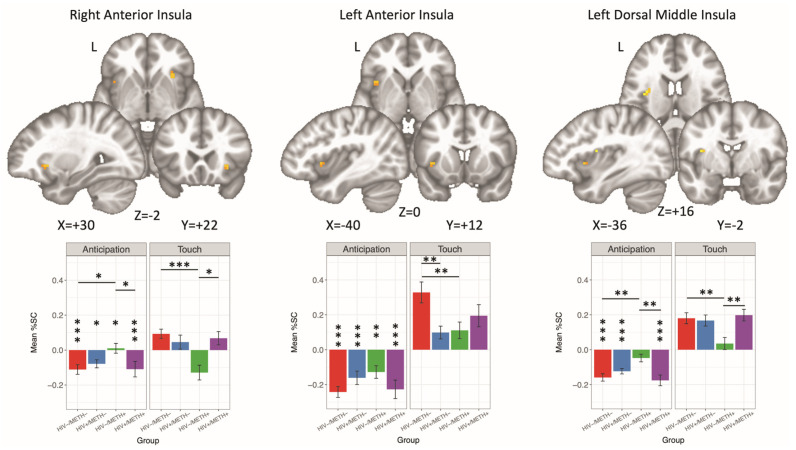
A significant HIV × METH × Condition interaction was detected in three separate clusters within the insula. For nearly all groups and clusters, participants had greater BOLD responses to touch receipt relative to touch anticipation (*p* value is denoted with vertical asterisks over anticipation bars). Within both the right anterior insula and left dorsal middle insula, both HIV−/METH− and HIV+/METH+ had significantly lower BOLD response to anticipation relative to HIV−/METH+, but greater BOLD response to touch receipt. Within the left anterior insula, HIV−/METH− had higher BOLD response to pleasant touch relative to the HIV+/METH− and HIV−/METH+ cohorts. %SC: percent signal change. * *p* < 0.05; ** *p* < 0.01; *** *p* < 0.001.

Three clusters showing significant HIV × METH × Condition interactions were identified (Table 2, Figure 4). Within the bilateral anterior insula and the left dorsal middle insula, the HIV−/METH−, HIV+/METH−, and HIV+/METH+ cohorts demonstrated a stronger BOLD response to touch relative to anticipation. In contrast, the HIV−/METH+ group demonstrated this effect only within the left anterior insula, while within the right anterior insula the HIV−/METH+ group showed a greater BOLD response to anticipation relative to touch. Differences stratified by either HIV serostatus or METH use disorder history were also identified for both anticipation and receipt. Within HIV seronegative groups, METH+ exhibited greater BOLD responses to anticipation within the left dorsal middle insula, while METH− responded more strongly to touch receipt within the bilateral anterior insula and left dorsal middle insula. For METH+ groups, individuals who were seronegative for HIV responded more strongly to anticipation within the right anterior insula as well as the left dorsal middle insula, whereas PWH showed a greater BOLD response to touch receipt within the right anterior insula and left middle dorsal insula. Finally, within METH− groups, HIV seronegative individuals exhibited greater BOLD response to pleasant touch within the left anterior insula relation to HIV seropositive individuals. There were no significant findings for the HIV × METH interaction, nor were there significant main effects of either HIV or METH.

### 3.5. Associations with Clinical Variables

#### 3.5.1. PWH

**Figure 5:**
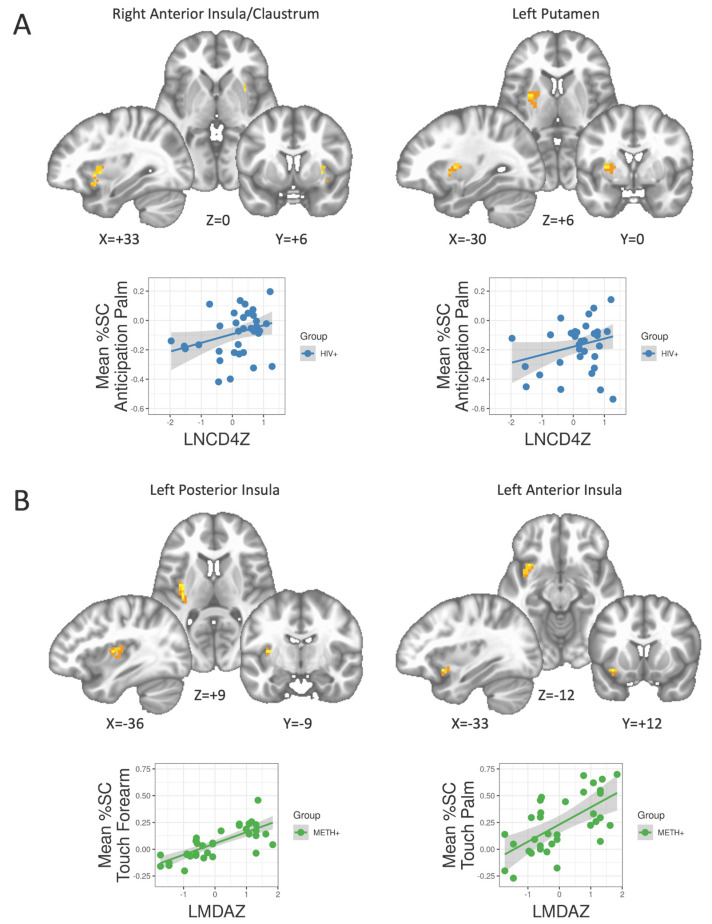
Clusters within insula and striatal regions with significant relationships to HIV or METH related clinical variables. (**A**) HIV+ individuals with lower CD4 nadir had lower BOLD responses to anticipation of touch in both the right anterior insula and left putamen. Figure 5. (**B**) METH+ participants with longer periods of abstinence had greater BOLD response to pleasant touch in the left anterior and posterior insula. %SC: percent signal change; LMDAZ: z-score of the log of days abstinent; LNCD4Z: z-score of the log of Nadir CD4.

Huber robust regression suggested that lower CD4 nadir was associated with lower BOLD response within the right anterior insula, extending into the claustrum, to the anticipation of soft touch of the palm, *t*(34) = 1.72 (Figure 5A). Similarly, lower CD4 nadir was associated with a lower BOLD response within the left putamen to the anticipation of soft touch of the forearm, *t*(34) = 1.42.

#### 3.5.2. Individuals with a History of METH Use Disorder

Longer abstinence (as measured by days since last use) was associated with a higher BOLD response the left posterior insula to pleasant touch of the palm, *t*(34) = 7.22 (Figure 5B). A separate cluster within the left anterior insula also suggested that longer abstinence was associated with a higher BOLD response to pleasant touch of the forearm, *t*(34) = 4.25.

## 4. Discussion

The present study suggests that PWH with no METH use disorder history exhibit altered neural signals within the insula, a region involved in predicting and interpreting pleasant interoceptive stimuli in comparison to HIV−/METH− individuals. However, METH use disorder history in PWH did not appear to introduce additional interoceptive or reward processing impairment relative to PWH with no METH use disorder history. Rather, PWH with METH use disorder history were not statistically different in terms of touch anticipation and receipt relative to non-using seronegative controls, suggesting that the interaction of HIV infection with METH is not additive. Taken together, these findings support disrupted interoceptive processing, but additionally suggest this effect is minimized in PWH with histories of METH use disorder.

### 4.1. Significance of Findings

Overall, we did not find significant neural activation differences related solely to HIV status that were independent of METH use disorder history. Rather, for individuals without METH use disorder histories, PWH exhibited reduced neural activation to soft touch receipt in the left anterior insula relative to seronegative controls. Neuroanatomical studies have reported reduced cortical volume in PWH relative to seronegative controls in multiple regions, including the insula [69] and is further exacerbated in association with HIV-associated distal neuropathic pain in PWH [70,71,72]. Others have reported reduced intrinsic resting state activity within the insula in PWH, [73] and, more broadly, reduced functional connectivity between the insula and other regions involved in the salience network [74,75,76,77]. More recent work has reported increased right anterior insula activation to expectation of pain offset PWH with distal neuropathic pain relative to PWH without this condition [78], suggesting abnormal processing of pain relief in those with chronic pain. Our own work has reported greater activation in the anterior insula to risky decisions in PWH relative to HIV seronegative individuals [9]; however, this could have been due to increased activation in the caudate nucleus, anterior cingulate, and dorsolateral prefrontal cortex, as these regions are reciprocally connected with the anterior insula [79]. An attenuated response to soft touch could reflect changes in interoceptive awareness. Although PWH, regardless of METH history, reported similar levels of pleasantness relative to seronegative controls, they also reported that pleasant touch felt more intense. Our findings therefore suggest that there could be changes in the interpretation of affective touch that predate neurological symptoms, as the majority of our participants did not meet the threshold for distal polyneuropathy. Additional research is needed to better address this question.

We did detect a two-way interaction of METH use disorder history with Condition, whereby METH+ individuals, regardless of HIV status, demonstrated an attenuated response to soft touch receipt within the left posterior insula, right anterior insula, left dorsal caudate, and left posterior putamen relative to non-using individuals. METH has consistently been linked to deficits in striatal functioning to reward processing and risk behavior [12,80,81], as well as to interoceptive deficits in the insula [29,82,83]. Moreover, others have reported reduced insula activation during other tasks, including cognitive control [84] and decision making [85]. Our findings are consistent with the broader literature of attenuated response to pleasant stimuli in the striatum, supporting a general thesis that METH is associated with an impaired ability to process reward, along with an attenuated insula response that is indicative of an impaired ability to predict the bodily experience to exteroceptive stimuli.

Our results also demonstrated that prior METH use disorder history may interact with HIV serostatus, suggesting a possible synergistic effect on BOLD activation within the insula. The HIV−/METH+ group demonstrated reduced BOLD response to pleasant touch in the bilateral anterior insula and left dorsomedial insula. A similar relationship was found for METH− individuals, as HIV infection was associated with a lower BOLD response in the left anterior insula. However, for individuals with METH use disorder histories, PWH had greater responses to pleasant touch in the right anterior insula relative to seronegative individuals. Moreover, the HIV+/METH+ group was not statistically different from the HIV−/METH− group. Prior work examining brain morphometry has reported smaller cortical and subcortical volume and thinner cortex in PWH relative to seronegative controls, and modest reductions in cortical thickness in individuals with prior METH history, yet no significant differences in their interaction [15,16]. Similarly, others have reported nonsignificant interactions of METH and HIV infection in brain metabolites [13,86] or cerebral blood flow [14] although it has been suggested that effects of HIV and METH could be additive [13,14].

In contrast, others have reported that while main effects of HIV or METH during a complex motor task were associated with attenuated BOLD responses in the striatum and insula, the BOLD activation in the HIV+/METH+ group was more similar to HIV−/METH− [17]. In seronegative individuals, active METH use increases extracellular dopamine due to the reduction of dopaminergic transporters, increases glutamate levels, and activates inflammatory pathways [30]. HIV infection has also been associated with elevated levels of glutamate [87], impaired dopaminergic functioning [88], and inflammation. Little is known about the interaction of METH use disorder with HIV infection on inflammation, although evidence suggests it could be exacerbated, as METH-using PWH show greater cognitive impairment [89]. Both HIV infection and METH use affect the peripheral immune system, typically by elevating inflammatory cytokines and chemokines [90,91], and the comorbid use of METH in the context of HIV may additionally impact immune system homeostasis. Moreover, dopamine also regulates immune functioning, and immune systems can subsequently influence dopamine signaling [92], thereby modulating tactile perception and perceived intensity [93]. However, prolonged abstinence from METH in seronegative individuals has also been shown to largely normalize glutamate levels [94], as well as promote significant recovery of dopaminergic transporters in the brain [95]. As METH abstinence has been linked to lower viral loads in PWH [96], this could suggest some reduction of excess glutamate, which is associated with inflammation, along with less dysregulation of dopaminergic systems. For the purpose of pleasant touch, it is possible that METH abstinence in PWH might lead to modest improvements in dopamine and glutamate function, allowing for neural activity that appears more similar to non-using seronegative controls. Others have suggested that improvements related to prolonged abstinence from METH use are specific to younger PWH, as the combination of prior neurotoxicity coupled with the aging process might be especially impactful [97]. More comprehensive studies are needed to address this question.

Within all PWH, there was a modest association of lower CD4 nadir with lower BOLD responses to soft touch anticipation in the right anterior insula and left putamen. Other studies have linked lower nadir CD4 to altered risk-related neural processing [9,98]. Lower CD4 nadir has also been linked to cortical thinning and reduced cortical gray matter volume [16,99,100,101], although others have reported no associations [102,103]. Our findings provide further support for associations between historical immunocompromise and vulnerability of HIV-related neural injury [104], including for interoception. Among individuals with METH use disorder histories, longer abstinence was associated with higher activation in the insula to receipt of pleasant touch. Other studies of interoceptive processing have not found this association; however, those studies were in recently abstinent individuals [29], suggesting that longer periods of abstinence could lead to improvements in interoceptive neural processing.

### 4.2. Limitations

This study has several limitations. It is not possible to determine whether findings are related to premorbid traits or whether findings are a consequence of METH exposure or presence of HIV infection. It is also possible that the initiation of METH use relative to HIV seroconversion might play a role [105]. Although our analyses were focused on METH use disorder history in the context of HIV infection, we did not exclude participants for marijuana, alcohol, or nicotine use, as these substances often overlap with METH use. It is possible that MRI eligibility and willingness to participate may have induced sampling bias due to a relatively healthier study population. This study also assessed the anticipatory response to predictable and certain events, and it’s possible the level of predictability may have influenced the anticipatory response. The inclusion of a continuous performance task and an anticipatory phase may also have mitigated our ability to detect meaningful differences related to C-fiber stimulation. Attention [106] and other contextual information, such as visual stimuli or one’s internal motivational state, [107,108,109] can modulate one’s sensory awareness. Finally, smaller sample size, particularly for the HIV+/METH+ group, could mean our results are not generalizable, and larger studies are needed.

## 5. Conclusions

The present study examined the independent and combined effects of HIV infection and methamphetamine use disorder history on pleasant touch, an interoceptive process. We found that while METH use disorder history, regardless of HIV status, attenuated insular and striatal response to pleasant touch, a reduced response to pleasant touch in PWH was only seen in the insula for the non-using cohort. Finally, HIV+/METH+ individuals did not show statistically significant differences in neural activation from METH− seronegative controls. These findings suggest that METH and HIV may differentially affect interoceptive processing.

## Figures and Tables

**Table 1:** Demographic and clinical characteristics of participants.

Characteristic	HIV−/METH− (N = 28)	HIV+/METH− (N = 20)	HIV−/METH+ (N = 19)	HIV+/METH+ (N = 16)	*p*-Value
Age (years)	38.8 (11.2)	40.1 (11.3)	37.2 (8.66)	41.5 (10.1)	NS
Education (years)	14.1 (2.05)	13.8 (2.47)	12.4 (2.31)	13.4 (1.78)	0.02
Male/Female ^☨^	24/4	18/2	18/1	16/0	NS
Handedness Right/Left ^☨^	27/1	17/3	18/1	16/0	NS
Ethnicity (% Caucasian) ^☨^	64	50	53	63	NS
Wide Range Achievement Test-4 Standard Score	53.79 (9.12)	49.55 (6.90)	49.16 (7.53)	53.88 (8.06)	0.015
Distal Symmetric Polyneuropathy (n) ^☨^	2	6	2	7	0.01
Loss of Sensation (n) ^☨^	0	3	0	4	0.004
Paresthesia (n) ^☨^	0	4	1	5	0.003
Dysesthesia (n) ^☨^	1	1	1	4	NS
Neuropathic pain (n) ^☨^	1	1	1	4	NS
Beck Depression Inventory II ^a^	2.36 (3.23)	7.70 (8.57)	13.2 (9.28)	12.4 (12.6)	<0.001
GE Signa/GE MR750 System ^☨^	10/18	3/17	11/8	6/10	0.05
**Methamphetamine Characteristics**					
Age of First Use			24.7 (8.36)	25.1 (7.80)	NS
Total Days Used			1934 (1511)	1720 (2269)	NS
Days Since Last Use ^a^			182 (160)	257 (190)	NS
Total Quantity (g)			2733 (3290)	1335 (2563)	NS
Use Density			1.28 (1.34)	0.75 (0.75)	NS
Primary Route of Use ^☨^			Smoking	Smoking	NS
**HIV Characteristics**					
Duration of Infection (months) ^b^		113.2 (100.6)		139.3 (106.5)	NS
Current antiretroviral use ^☨^		14		12	NS
Nadir CD4 Count		250.0 (170.5)		196.5 (171.2)	NS
Current CD4 Count ^c^		447.5 (283.2)		543.0 (259.5)	NS

Note: values in parenthesis are standard deviations. GDS: Global Deficit Score; NS: not significant. ^☨^ Fisher’s Exact Test; ^a^ Missing 1 HIV−/METH+, 2 HIV+/METH+; ^b^ Missing 1 HIV+/METH−; ^c^ Missing 1 HIV+/METH+.

**Table 2:** Regions of interest with significant BOLD activation for the Location (Palm, Forearm) + HIV × METH × Condition (Anticipation, Receipt) linear mixed effects analysis.

						Post Hoc Comparisons			
Structure	Volume (μL)	X	Y	Z	F(peak)	Contrast	t Ratio	*p*-Value	ES
	**MAIN EFFECT OF CONDITION**								
Left Insula Lobe	14769	−36	−12	4	133.42	Touch > Condition	11.67	<0.001	0.76
Right Insula Lobe	13716	36	16	−14	70.63	Touch > Condition	9.71	<0.001	0.56
Left Striatum	15849	−28	−8	−8	124.88	Touch > Condition	10.16	<0.001	0.71
Right Striatum	11880	12	−6	24	73.95	Touch > Condition	8.76	<0.001	0.51
	**HIV × CONDITION**								
Left Insula	108	−40	10	0	13.52	HIV−: Touch > Anticipation	9.25	<0.001	0.66
						HIV+: Touch > Anticipation	6.60	<0.001	0.53
	**METH × CONDITION**								
Left Posterior Insula	2322	−36	−12	4	22.57	METH−: Touch > Anticipation	15.44	<0.001	0.99
						METH+: Touch > Anticipation	8.75	<0.001	0.65
						Touch: METH− > METH+	3.20	0.003	0.22
Right Anterior Insula	1350	32	22	−6	26.53	METH−: Touch > Anticipation	9.55	<0.001	0.60
						METH+: Touch > Anticipation	3.01	0.004	0.22
						Anticipation: METH+ > METH−	2.26	0.03	0.15
						Touch: METH− > METH+	3.32	0.003	0.23
Left Anterior Insula	486	−28	24	12	18.10	METH−: Touch > Anticipation	6.76	<0.001	0.70
						METH+: Touch > Anticipation	2.49	0.03	0.48
Left Dorsal Insula	270	−34	0	16	21.74	METH−: Touch > Anticipation	12.66	<0.001	0.57
						METH+: Touch > Anticipation	7.38	<0.001	0.34
Left Ventral Anterior Insula	243	−30	16	−8	17.89	METH−: Touch > Anticipation	10.97	<0.001	0.74
						METH+: Touch > Anticipation	5.64	<0.001	0.55
Left Anterior Insula	189	−40	10	0	14.67	METH−: Touch > Anticipation	10.72	<0.001	0.66
						METH+: Touch > Anticipation	6.91	<0.001	0.35
Left Dorsal Caudate Nucleus	486	−16	4	16	15.92	METH−: Touch > Anticipation	12.21	<0.001	0.92
						METH+: Touch > Anticipation	5.59	<0.001	0.64
						Touch: METH− > METH+	3.36	0.002	0.21
Left Posterior Putamen/Claustrum	243	−34	−14	−2	14.32	METH−: Touch > Anticipation	13.29	<0.001	0.69
						METH+: Touch > Anticipation	8.01	<0.001	0.48
						Touch: METH− > METH+	2.82	0.01	0.15
Left Posterior Putamen	162	−28	−20	6	14.40	METH−: Touch > Anticipation	12.18	<0.001	0.65
						METH+: Touch > Anticipation	7.34	<0.001	0.41
						Touch: METH− > METH+	2.28	0.03	0.11
Left Ventral Striatum	135	−16	6	−12	14.25	METH−: Touch > Anticipation	9.29	<0.001	0.65
						METH+: Touch > Anticipation	5.00	<0.001	0.41
Left Posterior Putamen	135	−34	−6	4	18.44	METH−: Touch > Anticipation	12.15	<0.001	0.84
						METH+: Touch > Anticipation	7.42	<0.001	0.59
	**HIV × METH × CONDITION**								
Right Anterior Insula	189	30	22	−2	15.09	Anticipation, HIV−: METH+ > METH−	2.67	0.02	0.24
						Touch, HIV−: METH− > METH+	4.88	<0.001	0.44
						HIV−/METH−: Touch > Anticipation	5.08	<0.001	0.41
						HIV+/METH−: Touch > Anticipation	2.61	0.02	0.25
						HIV−/METH+: Anticipation > Touch	2.85	0.01	0.28
						HIV+/METH+: Touch > Anticipation	3.33	<0.001	0.35
						Anticipation, METH+: HIV− > HIV+	2.30	0.04	0.24
						Touch, METH+: HIV+ > HIV−	3.79	0.001	0.39
Left Anterior Insula	135	−40	12	0	13.10	Touch, HIV−: METH− > METH+	3.28	0.003	0.34
						HIV−/METH−: Touch > Anticipation	10.21	<0.001	0.90
						HIV+/METH−: Touch > Anticipation	3.93	<0.001	0.41
						HIV−/METH+: Touch > Anticipation	3.52	0.002	0.38
						HIV+/METH+: Touch > Anticipation	5.71	<0.001	0.66
						Touch, METH−: HIV− > HIV+	3.52	0.002	0.36
Left Dorsal Middle Insula	135	−36	−2	16	17.37	Anticipation, HIV−: METH+ > METH−	2.86	0.01	0.23
						Touch, HIV−: METH− > METH+	3.71	0.001	0.30
						HIV−/METH−: Touch > Anticipation	10.08	<0.001	0.71
						HIV+/METH−: Touch > Anticipation	7.30	<0.001	0.61
						HIV+/METH+: Touch > Anticipation	8.40	<0.001	0.78
						Anticipation, METH+: HIV− > HIV+	2.88	0.01	0.27
						Touch, METH+: HIV+ > HIV−	3.65	0.001	0.34

Note: Center of mass coordinates reported in MNI space. Small volume correction was determined with Monte-Carlo simulations (via AFNI’s 3dClustSim) to guard against false positives. ES: standardized effect size.

## Data Availability

Data will be made available on request.

## Supplementary Materials

The following are available online at https://www.mdpi.com/article/10.3390/v13122476/s1. Table S1: Statistical comparisons were by means of fisher-exact tests for equality of proportions; Table S2: Significant BOLD activation for the Location (Palm, Forearm) + HIV × METH × Condition (Anticipation, Receipt) linear mixed effects whole brain analysis.
