# Supplementary material for: Prior Methamphetamine Use Disorder History Does Not Impair Interoceptive Processing of Soft Touch in HIV Infection
Source: Viruses. 2021 Dec 10;13(12):2476. doi: 10.3390/v13122476 (PMC8705776; doi:10.3390/v13122476)
Supplement: Supplementary file 1 [file viruses-13-02476-s001.zip › viruses-1484741-supplementary.pdf]

Supplemental Table S1. Statistical comparisons were by means of fisher-exact tests for equality of proportions.

Characteristic	HIV-/METH-	HIV-/METH+	HIV+/METH-	HIV+/METH+	Statistic
**Lifetime Psychiatric Histories (n)**					
Antisocial Personality Disorder	1	10	3	3	FET, p<0.001
Attention-deficit/hyperactivity disorder	1	5	2	2	FET, p=0.13
Major depressive disorder	5	9	10	11	FET, p=0.006
**Substance Use Histories (n)**					
Current Nicotine Use	9	11	7	6	FET, p=0.33
Alcohol (lifetime)	12	16	5	8	FET, p=0.002
Cannabis (lifetime)	7	11	4	4	FET, p=0.05
Cocaine (lifetime)	0	8	0	3	FET, p<0.001
Opiates (lifetime)	0	2	0	1	FET, p=0.11

Supplemental Information: Prior Methamphetamine Use Disorder History Does Not Impair Interoceptive Processing of Soft Touch in HIV Infection

Amanda Bischoff-Grethe, Ph.D., Ronald J. Ellis, M.D., Ph.D., Susan F. Tapert, Ph.D., Martin P. Paulus, M.D., Igor Grant, M.D., Translational Methamphetamine AIDS Research Center (TMARC)

Supplemental Table S2. Significant BOLD activation for the Location (Palm, Forearm) + HIV x METH x Condition (Anticipation, Receipt) linear mixed effects whole brain analysis.

						Post hoc comparisons			
**Structure**	**Volume**	**X**	**Y**	**Z**	**F-value (peak)**	**Contrast**	**t ratio**	**p value**	**ES**
**MAIN EFFECT OF LOCATION**									
**R Postcentral Gyrus**	15093	38	-20	48	72.79				
**L Culmen**	1323	-22	-48	-24	21.28				
**MAIN EFFECT OF CONDITION**									
**L Thalamus**	744984	-30	-2	-20	258.17	Touch > Anticipation	15.33	<.001	0.49
**R Middle Temporal Gyrus**	13581	54	-66	6	233.32	Touch > Anticipation	15.06	<.001	0.50
**METH x CONDITION**									
**L Parahippocampal Gyrus**	94878	-12	-42	-6	30.25	METH-: Touch > Anticipation	13.33	<.001	0.61
						METH+: Touch > Anticipation	5.02	<.001	0.27
						Touch: METH- > METH+	4.57	<.001	0.27
**L Cingulate Gyrus**	12312	-30	36	30	39.72	METH-: Touch > Anticipation	6.49	<.001	0.32
						Touch: METH- > METH+	4.16	<.001	0.26
**L Medial Frontal Gyrus**	6939	-10	-8	72	31.03	METH-: Touch > Anticipation	4.30	<.001	0.23
						Touch: METH- > METH+	3.63	0.001	0.29
**R Insula**	2646	32	22	-6	26.53	METH-: Touch > Anticipation	11.11	<.001	0.62
						METH+: Touch > Anticipation	4.21	<.001	0.27
						Touch: METH- > METH+	3.44	0.001	0.22
**R Precuneus**	1647	6	-72	18	24.88	METH-: Touch > Anticipation	6.90	<.001	0.29
						Touch: METH- > METH+	3.40	0.002	0.24
**R Middle Frontal Gyrus**	1215	42	46	22	24.66	METH+: Anticipation > Touch	3.07	0.01	0.20
						Touch: METH- > METH+	2.43	0.04	0.25
**L Middle Frontal Gyrus**	1107	-34	-2	60	24.96	METH-: Touch > Anticipation	2.93	0.01	0.15
						Touch: METH- > METH+	2.83	0.01	0.19
**R Middle Frontal Gyrus**	999	30	10	46	17.65	Touch: METH- > METH+	3.06	0.01	0.19
**R Cingulate Gyrus**	945	8	-32	42	24.66	METH-: Touch > Anticipation	8.98	<.001	0.44
						METH+: Touch > Anticipation	2.44	0.02	0.14
						Touch: METH- > METH+	3.52	0.001	0.21
**L Insula**	891	-28	24	12	18.06	METH-: Touch > Anticipation	8.30	<.001	0.39

						METH+: Touch > Anticipation	3.11	0.004	0.17
**L Hypothalamus**	810	-4	-6	-14	23.07	METH-: Touch > Anticipation	12.92	<.001	0.79
						METH+: Touch > Anticipation	5.57	<.001	0.40
						Anticipation: METH+ > METH-	2.24	0.03	0.19
						Touch: METH- > METH+	2.40	0.03	0.21
**L Superior Frontal Gyrus**	702	6	-6	60	19.42	n.s.			
**R Inferior Frontal Gyrus**	486	50	12	24	20.86	METH-: Touch > Anticipation	3.37	0.004	0.18
**L Insula**	459	-30	16	-8	17.89	METH-: Touch > Anticipation	10.97	<.001	0.55
						METH+: Touch > Anticipation	5.77	<.001	0.33
**L Insula**	459	-36	22	6	16.27	METH-: Touch > Anticipation	10.64	<.001	0.61
						METH+: Touch > Anticipation	6.41	<.001	0.42
**R Superior Temporal Gyrus**	459	60	-6	10	22.18	METH-: Touch > Anticipation	9.38	<.001	0.55
						METH-: Touch > Anticipation	2.37	0.02	0.16
						Touch: METH- > METH+	4.12	<.001	0.38
**L Cuneus**	459	-10	-92	28	21.28	n.s.			
**L Middle Temporal Gyrus**	432	-46	-8	-18	18.8	METH-: Touch > Anticipation	9.19	<.001	0.54
						METH+: Touch > Anticipation	4.43	<.001	0.30
**L Insula**	432	-40	12	16	27.94	METH-: Touch > Anticipation	11.80	<.001	0.57
						METH+: Touch > Anticipation	5.58	<.001	0.31
						Anticipation: METH+ > METH-	2.96	0.005	0.16
**R Middle Frontal Gyrus**	432	20	-14	58	19.99	METH+: Anticipation > Touch	3.22	0.003	0.14
						Touch: METH- > METH+	3.56	0.003	0.19
**L Superior Temporal Gyrus**	405	-54	-36	10	19.96	METH-: Touch > Anticipation	7.73	<.001	0.33
						METH+: Touch > Anticipation	4.02	<.001	0.20
**R Parahippocampal Gyrus**	378	14	-2	-12	23.84	METH-: Touch > Anticipation	12.49	<.001	0.85
						METH+: Touch > Anticipation	2.59	0.01	0.20
						Touch: METH- > METH+	5.24	<.001	0.47
**R Superior Frontal Gyrus**	378	18	-12	72	27.13	METH+: Anticipation > Touch	4.24	<.001	0.35
						Touch: METH- > METH+	2.84	0.01	0.27
**R Superior Temporal Gyrus**	351	54	-56	18	17.08	METH-: Touch > Anticipation	3.21	0.003	0.14
						METH+: Touch > Anticipation	9.91	<.001	0.52
						Touch: METH+ > METH-	2.81	0.008	0.26
**R Postcentral Gyrus**	351	54	-14	18	22.80	METH-: Touch > Anticipation	13.24	<.001	0.58
						METH+: Touch > Anticipation	7.91	<.001	0.40
						Touch: METH- > METH+	2.58	0.02	0.21
**L Cingulate Gyrus**	324	-16	12	28	17.23	METH-: Touch > Anticipation	9.17	<.001	0.51

						METH+: Touch > Anticipation	3.71	0.001	0.24
						Touch: METH- > METH+	3.28	0.002	0.20
**R Middle Frontal Gyrus**	324	44	-2	52	20.82	METH+: Anticipation > Touch	4.05	<.001	0.24
**R Middle Frontal Gyrus**	324	20	-2	54	20.31	METH-: Touch > Anticipation	3.22	0.006	0.13
						Touch: METH- > METH+	3.06	0.006	0.17
**HIV x METH x CONDITION**									
**L Cuneus**	9774	-4	-86	16	30.84	HIV-, Touch: METH- > METH+	4.65	<.001	0.39
						HIV-, METH-: Touch > Anticipation	8.77	<.001	0.45
						HIV+, METH-: Touch > Anticipation	4.20	<.001	0.26
						HIV+, METH+: Touch > Anticipation	7.55	<.001	0.52
						METH+, Touch: HIV+ > HIV-	4.29	<.001	0.41
**L Superior Temporal Gyrus**	3321	-52	-44	12	29.34	HIV-, Touch: METH- > METH+	2.59	0.02	0.25
						HIV+, Touch: METH+ > METH-	3.80	<.001	0.41
						HIV-, METH-: Touch > Anticipation	8.23	<.001	0.45
						HIV+, METH-: Touch > Anticipation	3.92	<.001	0.25
						HIV+, METH+: Touch > Anticipation	10.55	<.001	0.76
						METH+, Touch: HIV+ > HIV-	4.94	<.001	0.54
**R Cingulate Gyrus**	2322	6	16	40	26.7	HIV-, Touch: METH- > METH+	4.47	<.001	0.37
						HIV+, Touch: METH+ > METH-	2.39	0.04	0.22
						HIV-, METH-: Touch > Anticipation	5.70	<.001	0.37
						HIV+, METH+: Touch > Anticipation	4.72	<.001	0.41
						METH+, Anticipation: HIV- > HIV+	2.27	0.04	0.22
						METH-, Touch: HIV- > HIV+	2.98	0.009	0.25
						METH+, Touch: HIV+ > HIV-	3.70	0.001	0.35
**L Inferior Parietal Lobule**	1863	-40	-56	48	24.48	HIV-, Touch: METH- > METH+	3.53	0.003	0.30
						HIV-, METH-: Touch > Anticipation	4.84	<.001	0.27
						HIV-, METH+: Anticipation > Touch	2.35	0.05	0.16
						HIV+, METH+: Touch > Anticipation	4.70	<.001	0.35
						METH+, Touch: HIV+ > HIV-	3.39	0.003	0.16
**L Middle Frontal Gyrus**	1809	-30	36	30	28.49	HIV-, Touch: METH- > METH+	5.20	<.001	0.44
						HIV+, Touch: METH+ > METH-	2.35	0.04	0.22
						HIV-, METH-: Touch > Anticipation	6.57	<.001	0.39
						HIV+, METH+: Touch > Anticipation	5.67	<.001	0.44
						METH-, Touch: HIV- > HIV+	2.38	0.04	0.20
						METH+, Touch: HIV+ > HIV-	4.83	<.001	0.47

**R Cuneus**	1053	12	-90	6	21.4	HIV+, Touch: METH+ > METH-	2.73	0.02	0.33
						HIV-, METH+: Anticipation > Touch	3.84	<.001	0.24
						HIV+, METH+: Touch > Anticipation	4.03	<.001	0.27
						METH+, Touch: HIV+ > HIV-	2.90	0.02	0.35
**L Fusiform Gyrus**	918	-42	-56	-6	24.15	HIV-, Anticipation: METH+ > METH-	2.83	0.01	0.26
						HIV-, Touch: METH- > METH+	2.77	0.01	0.26
						HIV-, METH-: Touch > Anticipation	9.14	<.001	0.59
						HIV+, METH-: Touch > Anticipation	4.65	<.001	0.35
						HIV+, METH+: Touch > Anticipation	8.32	<.001	0.71
						METH+, Anticipation: HIV- > HIV+	3.15	0.007	0.33
						METH+, Touch: HIV+ > HIV-	2.90	0.01	0.30
**L Culmen**	783	-6	-36	-24	18.86	HIV-, Touch: METH- > METH+	5.92	<.001	0.60
						HIV-, METH-: Touch > Anticipation	7.90	<.001	0.65
						HIV+, METH-: Touch > Anticipation	4.22	<.001	0.41
						HIV+, METH+: Touch > Anticipation	5.57	<.001	0.61
						METH+, Touch: HIV+ > HIV-	4.16	<.001	0.48
**R Posterior Cingulate**	702	18	-66	6	22.01	HIV-, Touch: METH- > METH+	3.26	0.007	0.35
						HIV-, METH-: Touch > Anticipation	5.73	<.001	0.32
						HIV+, METH+: Touch > Anticipation	5.11	<.001	0.38
**L Medial Frontal Gyrus**	702	0	-12	76	25.34	n.s.			0.00
**L Precentral Gyrus**	621	-48	10	0	21.74	HIV-, Touch: METH- > METH+	3.14	0.007	0.28
						HIV-, METH-: Touch > Anticipation	9.61	<.001	0.63
						HIV+, METH-: Touch > Anticipation	5.52	<.001	0.43
						HIV-, METH+: Touch > Anticipation	2.46	0.02	0.20
						HIV+, METH+: Touch > Anticipation	8.74	<.001	0.76
						METH+, Anticipation: HIV- > HIV+	2.96	0.01	0.30
						METH+, Touch: HIV+ > HIV-	2.61	0.02	0.26
**R Postcentral Gyrus**	594	60	-18	16	19.33	HIV-, Touch: METH- > METH+	4.00	<.001	0.46
						HIV+, Touch: METH+ > METH-	2.91	0.009	0.37
						HIV-, METH-: Touch > Anticipation	8.70	<.001	0.60
						HIV+, METH-: Touch > Anticipation	6.10	<.001	0.49
						HIV-, METH+: Touch > Anticipation	2.71	0.01	0.23
						HIV+, METH+: Touch > Anticipation	10.34	<.001	0.94
						METH+, Touch: HIV+ > HIV-	5.27	<.001	0.69
**L Declive**	567	-22	-60	-18	20.78	HIV-, Touch: METH- > METH+	3.38	0.003	0.35
						HIV-, METH-: Touch > Anticipation	9.59	<.001	0.63

						HIV+, METH-: Touch > Anticipation	6.83	<.001	0.53
						HIV+, METH+: Touch > Anticipation	8.01	<.001	0.69
						METH+, Anticipation: HIV- > HIV+	2.74	0.02	0.33
						METH+, Touch: HIV+ > HIV-	2.49	0.03	0.30
**L Middle Temporal Gyrus**	540	-46	-18	-14	17.5	HIV-, Anticipation: METH+ > METH-	3.20	0.005	0.29
						HIV-, Touch: METH- > METH+	2.28	0.04	0.20
						HIV-, METH-: Touch > Anticipation	8.37	<.001	0.56
						HIV+, METH-: Touch > Anticipation	4.08	<.001	0.32
						HIV+, METH+: Touch > Anticipation	7.90	<.001	0.69
						METH+, Anticipation: HIV- > HIV+	2.81	0.01	0.41
						METH+, Touch: HIV+ > HIV-	3.34	0.004	0.34
**L Postcentral Gyrus**	513	-48	-30	34	21.28	HIV-, Touch: METH- > METH+	2.97	0.009	0.25
						HIV-, METH-: Touch > Anticipation	7.06	<.001	0.37
						HIV+, METH-: Touch > Anticipation	4.54	<.001	0.28
						HIV+, METH+: Touch > Anticipation	7.44	<.001	0.52
						METH+, Touch: HIV+ > HIV-	3.37	0.004	0.32
**R Medial Frontal Gyrus**	486	26	48	16	15.52	HIV-, Touch: METH- > METH+	3.03	0.01	0.23
						HIV-, METH-: Touch > Anticipation	5.72	<.001	0.35
						HIV+, METH+: Touch > Anticipation	4.39	<.001	0.36
						METH-, Touch: HIV- > HIV+	2.88	0.02	0.21
**R Parahippocampal Gyrus**	405	8	-44	-2	26.12	HIV-, Touch: METH- > METH+	4.77	<.001	0.54
						HIV-, METH-: Touch > Anticipation	6.53	<.001	0.46
						HIV+, METH-: Touch > Anticipation	2.57	0.03	0.22
						HIV+, METH+: Touch > Anticipation	5.60	<.001	0.53
						METH+, Touch: HIV+ > HIV-	3.74	0.001	0.49
**R Precentral Gyrus**	351	56	6	12	22.00	HIV-, Touch: METH- > METH+	3.34	0.004	0.32
						HIV-, METH-: Touch > Anticipation	6.07	<.001	0.34
						HIV+, METH+: Touch > Anticipation	4.83	<.001	0.36
						METH+, Touch: HIV+ > HIV-	3.35	0.004	0.37
**R Supramarginal Gyrus**	351	44	-48	34	20.63	HIV+, Touch: METH+ > METH-	3.50	0.005	0.32
						HIV-, METH-: Touch > Anticipation	2.97	0.01	0.16
						HIV+, METH+: Touch > Anticipation	5.11	<.001	0.38
						METH-, Touch: HIV- > HIV+	2.62	0.03	0.21
						METH+, Touch: HIV+ > HIV-	2.95	0.01	0.27
**L Insula**	324	-36	0	16	19.25	HIV-, Anticipation: METH+ > METH-	3.09	0.005	0.24
						HIV-, Touch: METH- > METH+	3.59	0.001	0.28

						HIV-, METH-: Touch > Anticipation	10.58	<.001	0.71
						HIV+, METH-: Touch > Anticipation	7.98	<.001	0.63
						HIV-, METH+: Touch > Anticipation	2.30	0.03	0.19
						HIV+, METH+: Touch > Anticipation	9.22	<.001	0.82
						METH+, Anticipation: HIV- > HIV+	3.16	0.004	0.28
						METH+, Touch: HIV+ > HIV-	3.90	0.001	0.35
**L Inferior Parietal Lobule**	324	-42	-36	34	15.47	HIV-, Anticipation: METH+ > METH-	2.79	0.01	0.18
						HIV-, Touch: METH- > METH+	3.02	0.008	0.20
						HIV-, METH-: Touch > Anticipation	10.10	<.001	0.47
						HIV+, METH-: Touch > Anticipation	6.05	<.001	0.34
						HIV+, METH+: Touch > Anticipation	7.47	<.001	0.46
						METH+, Anticipation: HIV- > HIV+	3.22	0.006	0.24

Note: Center of mass coordinates reported in MNI space. Small volume correction was determined with Monte-Carlo simulations (via AFNI's 3dClustSim) to guard against false positives. ES: standardized effect size; L: left; R: right.
